# Unintentional Discontinuation of Statins May Increase Mortality After Traumatic Brain Injury in Elderly Patients: A Preliminary Observation

**DOI:** 10.4021/jocmr1333w

**Published:** 2013-04-23

**Authors:** Alessandro Orlando, David Bar-Or, Kristin Salottolo, Andrew Stewart Levy, Charles W Mains, Denetta S Slone, Patrick J Offner

**Affiliations:** a Trauma Research Department, St. Anthony Hospital, Lakewood, CO, USA; b Trauma Research Department, Swedish Medical Center, Englewood, CO, USA; c College of Osteopathic Medicine, Rocky Vista University, Parker, CO, USA; d InterMountain Neurosurgery, St. Anthony Hospital, Lakewood, CO, USA; e Trauma Services Department, St. Anthony Hospital, Lakewood, CO, USA; f Trauma Services Department, Swedish Medical Center, Englewood, CO, USA

**Keywords:** Statin, Discontinuation, Traumatic brain injury, Trauma, Elderly

## Abstract

**Background:**

The abrupt discontinuation of statin therapy has been suggested as being deleterious to patient outcomes. Although pre-injury statin (PIS) therapy has been shown to have a protective effect in elderly trauma patients, no study has examined how this population is affected by its abrupt discontinuation. This study examined the effects of in-hospital statin discontinuation on patient outcomes in elderly traumatic brain injury (TBI) patients.

**Methods:**

This was a multicenter, retrospective cohort study on consecutively admitted elderly (≥ 55) PIS patients who were diagnosed with a blunt TBI and who had a hospital length of stay (LOS) ≥ 3 days. Patients who received an in-hospital statin within 48 hours of admission were considered continued, and patients who never received an in-hospital statin were considered discontinued. Differences in in-hospital mortality, having at least one complication, and LOS > 1 week were examined between those who continued and discontinued PIS.

**Results:**

Of 93 PIS patients, 46 continued and 15 discontinued statin therapy. The two groups were equivalent vis-a-vis demographic and clinical characteristics. Those who discontinued statin therapy had a 4-fold higher mortality rate than those who continued (n = 4, 27% vs. n = 3, 7%, P = 0.055). Statin discontinuation did not have a higher complication rate, compared to statin continuation (n = 3, 20% vs. n = 7, 15%, P = 0.70), and no difference was seen in the proportion with a hospital LOS > 1 week (P > 0.99).

**Conclusions:**

Though our study is not definitive, it does suggest that the abrupt, unintended discontinuation of statin therapy is associated with increased mortality in the elderly TBI population. Continuing in-hospital statin therapy in PIS users may be an important factor in the prevention of in-hospital mortality in this elderly TBI population.

## Introduction

The majority of statin consumers are over the age of 50 [1] and simultaneously have a high risk of suffering a traumatic brain injury (TBI) [2]. In elderly trauma patients, pre-injury statin (PIS) therapy has been shown to have a protective effect [3-5]; nevertheless, the discontinuation of PIS therapy upon hospital admission has been recently suggested as having deleterious effects on patient outcomes. These studies examined cardiac [6, 7], stroke [8, 9] and vascular surgery populations [10], yet the elderly TBI population remains unexamined. The reasons for in-hospital statin discontinuation can be intentional, e.g. the patient is too severely injured to receive oral medications, or unintentional, e.g. clinical oversight. The objective of this study was to examine the extent to which unintentional statin discontinuation increases poor outcomes in the elderly TBI population. We hypothesized that unintentional statin discontinuation would be associated with worse patient outcomes, compared to continuation.

## Methods

### Study site and patient population

This study is a retrospective review of consecutively admitted adult trauma patients to two Level I Trauma Centers (first center: May 1, 2007 - November 20, 2008; second center: September 1, 2007 - August 31, 2009) with a blunt TBI which included at least one of the following diagnoses: cerebral contusion, subarachnoid hemorrhage or other unspecified intracranial hemorrhage, subdural or epidural hematoma, or diffuse axonal injury. Patients were excluded from the study because of no PIS use, age < 55 years, and a hospital length of stay (LOS) less than three days. This study was reviewed and approved by each institution’s Institutional Review Board.

Pre-injury statin use was identified as a statin medication in a patient’s paper chart or electronic medical record. If a PIS patient was never administered an in-hospital statin, they were considered to be discontinued from statin therapy. If a PIS patient’s in-hospital medications included the administration of a statin, they were considered to be continued on statin therapy.

**Figure 1:**
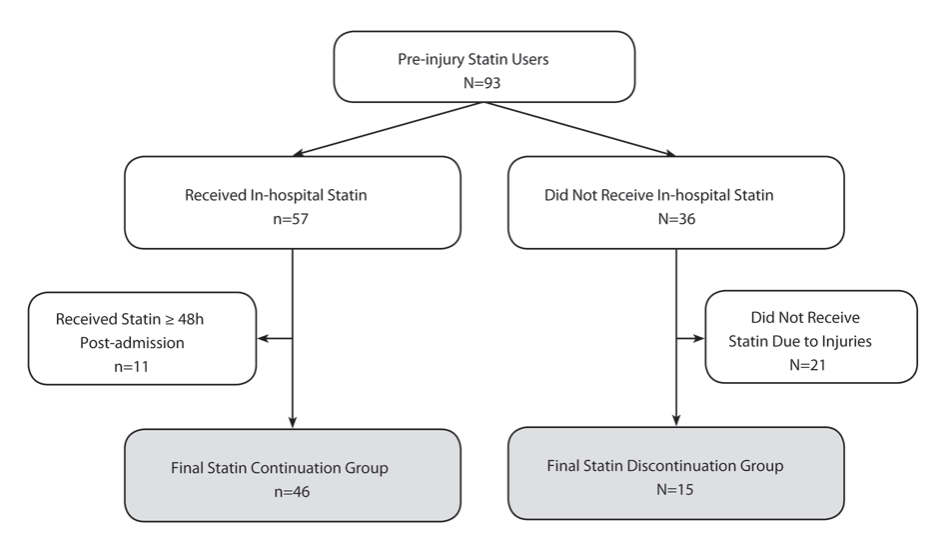
Study population flow-chart.

It is likely that severe injuries prevent some patients from being continued on an in-hospital statin. Therefore, independent, qualified hospital physicians and nurses reviewed the medical charts of all patients who were discontinued. Patients who were found to be intentionally discontinued from statin therapies within 48 hours of hospital admission for injury-related reasons (e.g. care withdrawal or intubation) were excluded to avoid bias (Fig. 1). Only those patients who were discontinued due to clinical oversight were included in the discontinuation group. There was no difference between hospitals in the rate of discontinuation due to clinical oversight (P = 0.74). Furthermore, we ensured that the cause of death was not related to a failure to comply with preventative care protocols (for example, deep vein thrombosis prophylaxis). If a do not resuscitate (DNR) protocol was activated in a patient with a likely survivable condition, that patient was excluded from the study.

### Outcomes and covariates

The primary outcome of this study was in-hospital mortality. Secondary outcomes were having at least one in-hospital complication, and a hospital LOS > 1 week. Covariates were defined as follows: age (years 55 - 79, ≥ 80), ED Glasgow Coma Scale (GCS, 3 - 12, 13 - 15), injury severity score (ISS, 0 - 15, ≥ 16), ED hypotension (systolic blood pressure < 90 mmHg), ED respiratory rate (abnormal (< 10 or > 29 breaths/minute), normal (10 - 29)), ED tachycardia (> 100 beats per minute), body mass index (BMI, normal (18.5 - 24.9 kg/m^2^) and underweight (16.5 - 18.4), vs. overweight (25 - 29.9) and obese (≥ 30)), mechanism of injury (fall, motor-vehicle accident and other), gender, pre-injury blood thinner use (clopidogrel, aggrenox, aspirin or warfarin), blood product transfusion (including fresh frozen plasma, packed red blood cells and albumin), Trauma Injury Severity Scale (TRISS) and the Charlson Comorbidity Index (CCI).

### Statistical analysis

Significant changes in inflammatory markers [11-15], and increased associations with mortality [7, 9] have been reported within 24 - 72 hours of statin therapy discontinuation. We chose 48 hours as the cutoff time within which patients had to have their in-hospital statin therapy continued. Based on the literature, we felt 48 hours was the average time after which we should expect to see statin discontinuation effects. Thus, we compared outcomes between patients who discontinued statin therapy upon hospital admission and those who continued statin therapy within 48 hours of hospital admission.

Pearson or Fisher’s exact chi-square tests were used to examine the effects of statin discontinuation on in-hospital mortality, the development of any complications and having a hospital LOS > 1 week. Only patients who survived to hospital discharge were included in the LOS analysis. All statistical analyses were performed using SAS® software, version 9.1 (SAS Institute, Cary, NC), and had an alpha value of 0.05.

## Results

A total of 93 patients were on PIS therapy, and the statin discontinuation rate was 38% (n = 36). After exclusion criteria were applied, the final study population consisted of 61 patients; 46 statin continuation and 15 statin discontinuation patients (Fig. 1). The most commonly prescribed in-hospital statin therapy was simvastatin (n = 27, 59%), followed by atorvastatin (n = 12, 26%), lovastatin (n = 5, 11%), pravastatin (n = 1, 2%) and rosuvastatin (n = 1, 2%). Both statin continuation and discontinuation groups were predominately male, had normal vital signs upon admission, mild head injuries, high ISS and TRISS survival probabilities, and similar CCI scores (Table 1). Body mass index was the only significant demographic difference between study groups; the statin continuation group had a significantly higher proportion of overweight or obese patients (P = 0.01).

**Table 1:** Patient Demographics Stratified by In-Hospital Statin Therapy Continuation^a^

Characteristics	Continued (n = 46)	Discontinued (n = 15)	P Value
Age			0.75
55 to 79	30 (65%)	11 (73%)	
≥ 80	16 (35%)	4 (27%)	
Male	25 (53%)	10 (67%)	0.40
BMI overweight or obese (≥ 25 kg/m^2^)	29 (67%)	4 (29%)	0.01
Pre-injury blood thinner use	40 (87%)	10 (67%)	0.12
ED GCS 13 - 15	37 (93%)	12 (92%)	> 0.99
ISS 0 - 15	16 (35%)	5 (33%)	0.92
ED hypotension (< 90 mmHg)	1 (2%)	0	n/a
ED respiratory rate (breaths/min)			0.42
< 10 or > 29	1 (2%)	1 (7%)	
10 to 29	44 (98%)	13 (93%)	
ED tachycardia (> 100 bpm)	7 (16%)	3 (21%)	0.69
TRISS, probability of survival ≥ 80%	35 (92%)	12 (92%)	> 0.99
Charlson comorbidity index			0.32
2 to 3	7 (15%)	4 (27%)	
4 to 6	30 (64%)	10 (67%)	
≥ 7	10 (21%)	1 (7%)	
Mechanism of Injury			0.18
Fall	37 (80%)	12 (80%)	
Motor vehicle or bicycle	8 (17%)	1 (7%)	
Other	1 (2%)	2 (13%)	
Blood product transfusion	11 (23%)	3 (20%)	0.72
ED ECG results			> 0.99
Abnormal	27 (75%)	10 (77%)	
Borderline	4 (11%)	1 (8%)	
Normal	5 (14%)	2 (15%)	
ED disposition			0.70
Direct admission	4 (9%)	1 (7%)	
General floor	9 (20%)	2 (13%)	
Intensive care unit	31 (67%)	10 (67%)	
Step-down unit	2 (4%)	2 (13%)	

^a^Not all data will sum to their respective totals due to missing data. BMI: body mass index; ED: emergency department; GCS: glasgow coma; ISS: injury severity score; BPM: beats per minute; TRISS: trauma and injury severity score; ECG: electrocardiogram.

The overall mortality rate was 11% (n = 7). The statin discontinuation group had a mortality rate 4-fold higher than the statin continuation group, trending toward statistical significance (n = 4, 27% vs. n = 3, 7%, P = 0.055). Due to the small sample size, we did not adjust for any variables; though, there were no significant demographic or clinical characteristics between study groups, except BMI (P = 0.01). The chart review revealed that 6 of the 7 deaths had an attached DNR; however, all DNRs were activated because of very poor expected outcomes and unlikely recoveries. Furthermore, no patient died from a failure to comply with a preventative care protocol.

The complication rates were similar between the two study groups (n = 3, 20% vs. n = 7, 15%, P = 0.70). In the discontinuation group, there were six complications across the three patients, and in the discontinuation group, there were 18 complications across the seven patients. The majority of complications were infection-related in both study groups (continuation: 37%; discontinuation: 67%). There was no significant difference in the proportion of patients with a LOS > 1 week between statin discontinuation and continuation groups (n = 3, 18% vs. n = 11, 23%, P > 0.99).

## Discussion

**Figure 2:**
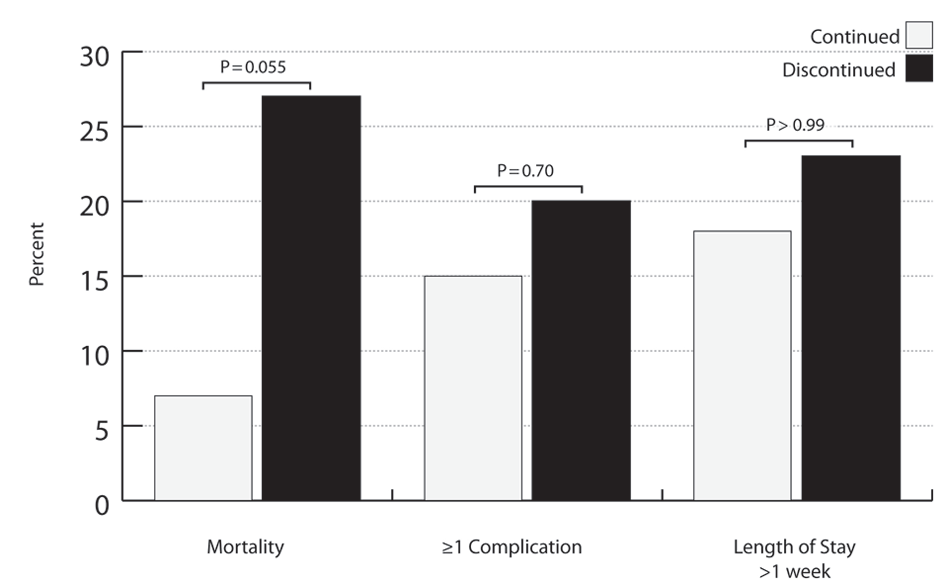
Outcomes between pre-injury statin continuation and discontinuation groups.

Physicians have long known the dangers associated with the sudden discontinuation of beta blockers, and much like beta blockers, the sudden discontinuation of statin therapy has also been suggested as being detrimental to patients’ health [6-10, 16-18]. Our study presents preliminary data that are in agreement with this theory and expands the affected population to include elderly TBI patients. Patients in our study who had unintended discontinuation of PIS had a mortality rate that trended towards being statistically significantly higher than those patients who were continued within 48 hours of admission (Fig. 2) (P = 0.055).

Two large studies examining statin therapy continuation have demonstrated that statin discontinuation is associated with higher mortality rates. Spencer et al. studied PIS use in over 19,500 patients across 14 countries with acute coronary syndrome [7]. They found that patients who had their statin therapy discontinued had a significantly higher mortality rate compared to those who continued (12% vs. 2%, P < 0.001). In another study, Spencer et al. examined a subset of patients from the National Registry of Myocardial Infarction and investigated the effects of PIS use in over 78,000 patients with myocardial infarction [6]. After adjustment, discontinuation of statin therapy was associated with a significant increase in the risk of mortality compared to patients who were continued (HR = 1.83, 95% CI: 1.58 to 2.13). Neither of these large-scale studies discounted the possibility that injury severity was associated with discontinuation and contributed to the increased mortality rate.

Meldrum and Moore et al. have presented compelling evidence suggesting that an initial insult, such a traumatic injury, primes the immune system for response, and a second event within a vulnerable time period may stimulate systemic inflammation [19, 20]. This two-event construct may explain why statin discontinuation, versus continuation, trended towards having a significantly increased mortality rates in our study and in other patient populations [6, 8, 9, 16, 17]. In our study, the TBI can be considered the first event, and the discontinuation of statin therapy might have acted as the second event. This theory is further reinforced by studies documenting changes in inflammatory markers following statin discontinuation, including decreased nitric oxide production, increased C-reactive protein and vascular cell adhesion molecule-1 levels [11, 12, 15].

Necessary steps must be taken to identify and separate patients who were discontinued from statin therapy because of their injuries, from patients who were discontinued because of clinical oversight. Patients who are discontinued from statin therapy because their care has been withdrawn are expected to have a higher risk of mortality than patients who are capable of receiving an oral statin medication but were not prescribed one. Thus, the discontinuation of statin therapy can be thought of as an indication of the severity of a patient’s injuries, and if not removed, will bias all outcome analyses [21]. Our study is unique in that it differentiated these discontinuation groups through a thorough chart review, ensuring that all instances of discontinuation were not related to events or indications that are associated with an increased mortality rate.

A full chart review might be impractical in large studies; however, the following variables would be beneficial in determining whether the severity of injury prevented the continuation of in-hospital statin therapy: nil per os, care withdrawal, DNR activation, receiving palliative care, intubation, coma, and emergent intervention. Furthermore, it is important to establish the temporality of the aforementioned variables. While some complications may prevent a patient from receiving in-hospital statins if they occur immediately after admission, other complications may be a consequence of not receiving an in-hospital statin. More importantly, the literature is void of a substantiated definition of statin withdrawal. Some studies illustrate deleterious effects after 24, 48 and 72 hours of statin discontinuation; our study chose a cut off value of 48 hours to define statin withdrawal. Future studies should aim to better understanding cellular and physiological changes subsequent to discontinuation.

The main limitation of our study was also one of its strengths: though our study had a small sample size, it made a thorough chart-review pragmatic. This allowed us to eliminate the possibility that statin therapy was discontinued because of a patient’s injuries, and to ensure that all DNRs were activated in patients who had deteriorated beyond recovery. However, the small sample size limited our ability to adjust for possible confounding factors, and resulted in our study being underpowered to detect the differences we observed.

We examined our data for variables that might introduce bias. In Table 1, we found three variables with a P < 0.20: BMI, pre-injury blood thinner use and mechanism of injury. However, when we examined the associations between these variables and mortality, we discovered that none of them had a P < 0.20; therefore, these variables are not likely to severely confound our mortality analysis. Generalizability is limited because our two Level I Trauma Centers’ standards of practice might differ from other hospitals. Despite our study’s limitations, the demographic and clinical similarities between study groups, combined with the exclusion of patients who were discontinued from statins because of severe injuries, lead us to believe that our univariate analyses were not highly influenced by differences between study groups.

### Conclusions

Though our study is not definitive, it does suggest that the abrupt, unintended discontinuation of statin therapy is associated with increased mortality in the elderly TBI population. The US Census Bureau estimates that the population aged 55 years and older will grow by 21 million over the next two decades [22]. It is likely that this population will continue to be a large consumer of statin medications, therefore our understanding of these patients and common medication usage must grow accordingly. Ensuring that a patient’s PIS use is communicated between the ED physicians and nurses, and those in the admitting hospital unit might be a simple, yet important factor in the prevention of elderly TBI mortality.
